# Development validation and reliability of the Systemic Lupus Erythematosus Disease Activity Index 2000 Responder Index-50

**DOI:** 10.1186/ar3989

**Published:** 2012-09-27

**Authors:** Z Touma, DD Gladman, MB Urowitz

**Affiliations:** 1 University of Toronto, ON, Canada

## Background

The Systemic Lupus Erythematosus Disease Activity Index 2000 (SLEDAI-2K) is a global disease activity index composed of 24 descriptors reflecting nine organ systems. The SLEDAI-2K measures only complete recovery in active descriptors on follow-up visits. Thus, an improvement in the SLEDAI-2K descriptors is captured when a manifestation has completely resolved. The aims were: to develop and validate an outcome measure, the SLEDAI-2K Responder Index-50 (S2K RI-50), able to capture partial improvement, ≥50%, in each of the active descriptors at subsequent visits; to test the reliability of S2K RI-50; and to evaluate S2K RI-50 sensitivity to response at 6 and 12 months.

## Methods

The S2K RI-50 was constructed based on SLEDAI-2K. New definitions for all descriptors were generated along with criteria to identify 50% improvement. The S2K RI-50 Data Retrieval Form standardizes the documentation of the descriptors. From September 2009 to April 2011, all lupus patients seen at the Lupus Clinic were assessed at baseline and follow-up visits using SLEDAI-2K, S2K RI-50 and physician global assessment (visual analog scale online and Likert scale). Concurrent construct validity: correlation of the S2K RI-50 was determined using the Likert scale as the external construct. Inter-rater/intra-rater reliability (intraclass correlation coefficient (ICC)): 40 patient scenarios with baseline and follow-up visits derived from real patients were developed. Ten rheumatologists scored the profiles with the SLEDAI-2K and S2K RI-50, on two occasions 2 weeks apart. Sensitivity to response: responders using the SLEDAI-2K, S2K RI-50 and SLE Responder Index (SRI) were identified at 6 and 12 months among 103 consecutive active patients. Patients defined as S2K RI-50 responders were compared with SRI responders; considered the gold standard.

## Results

The S2K RI-50 and its Data Retrieval Form were developed. The initial validation on 141 patients showed that S2K RI-50 has construct validity (correlated with Likert scale; *r *= 0.48; *P *< 0.0001). The S2K RI-50 is reliable and the ICC for inter-rater/intra-rater ranged from 0.86 to 1.00. The percentage of responders at 6 and 12 months was 44% and 51% by SLEDAI-2K and 43% and 51% by SRI respectively. The percentage of S2K RI-50 responders at 6 and 12 months was higher at 51% and 58% respectively.

## Conclusion

The S2K RI-50 is novel valid and reliable responder index able to identify patients with partial, ≥50% improvement. The S2K RI-50 identified more responders as compared with the SLEDAI-2K and SRI at the 6 and 12 month period. The S2K RI-50 can be used independently to identify patients with clinically important improvement.

